# Synthetic Homing Endonuclease Gene Drives to Revolutionise Aedes aegypti Biocontrol - Game Changer or Pipe Dream?

**DOI:** 10.1016/j.cois.2025.101373

**Published:** 2025-04-08

**Authors:** Joshua X.D. Ang, Sebald A.N. Verkuijl, Michelle A.E. Anderson, Luke Alphey

**Affiliations:** 1 The Department of Biology, University of York, Wentworth Way, York, YO10 5DD, U.K.; 2 York Biomedical Research Institute, University of York, Heslington YO10 5DD, U.K.; 3 Department of Life Sciences, Faculty of Natural Sciences, Imperial College London, U.K.

## Abstract

The increasing burden of *Aedes aegypti*-borne diseases, particularly dengue, is a growing global concern, further exacerbated by climate change. Current control strategies have proven insufficient, necessitating novel approaches. Synthetic homing endonuclease gene (sHEG) drives represent one of the few emerging technologies with the potential to offer a cost-effective and equitable solution to this escalating public health challenge. However, despite multiple attempts, the homing efficiencies of *Ae. aegypti* sHEG systems lag behind those achieved in *Anopheles* mosquitoes. We discuss key insights from efforts to develop sHEGs in *Ae. aegypti* and highlight critical factors that may unlock further advances in this species.

## Introduction

In recent years, synthetic homing endonuclease gene (sHEG) drives have gained considerable attention for their potential as cost-effective, species-specific tools for mosquito vector control^1–7^, potentially capable of invading populations following small initial introductions. In nature, HEGs function by inserting themselves into a specific genomic locus, functioning as an endonuclease that cleaves DNA at the same locus on the homologous chromosome^8^. This cleavage triggers DNA repair, during which the uncut chromosome—containing the HEG DNA sequence—serves as a repair template. The HEG is thereby copied to the cut and previously non-HEG-bearing chromosome, converting the cell from hemizygous to homozygous for the HEG. This process, termed homology-directed repair (HDR), is referred to as homing in the context of HEGs. When this conversion occurs in the germline cells of metazoans, the resulting gametes all carry the HEG, deviating from the 50% inheritance rate normally expected from a hemizygote. This mechanism could drive a beneficial genetic trait via this super-Mendelian inheritance pattern, enabling the alteration of wild mosquito populations by release of a small amount of sHEG-carrying mosquitoes^8^.

Since the development of the first sHEG in *Anopheles gambiae*^9^—the primary malaria vector in sub-Saharan Africa—research has progressed to testing sHEG strains in laboratory settings that replicate field conditions to evaluate their efficacy in mosquito control^10,11^. Discussions around the practical implementation of the first field trials have also been considered^12^. Leading global research collaborations, such as Target Malaria, have developed strategies to reduce or eliminate *An. gambiae* populations [“population suppression”]^4^, while others, such as Transmission Zero and the University of California Malaria Initiative (UCMI), aim to make the population refractory to malaria infection^13,14^ without substantial change in mosquito numbers [“population modification”]. Inspired by these successes, researchers have sought to replicate similar outcomes in other agricultural and vector pest species. Success in sHEG-based systems is typically assessed based on two critical criteria: (a) high super-Mendelian inheritance rates (“homing efficiency”) and (b) low fitness costs. Despite years of effort and numerous iterations, the inheritance biases induced in non-*Anopheles* insects (e.g. *Ceratitis capitata, Culex quinquefasciatus, Aedes aegypti, Plutella xylostella*) have significantly lagged behind those in *An. gambiae*^15–25^. The trend was largely similar for *Drosophila melanogaster*, except for one study, where homing was close to 100%^26–29^.

In this review, we focus on discussing the feasibility of developing a sHEG system in *Ae. aegypti. Ae. aegypti* is medically important as the key vector of dengue, chikungunya, yellow fever, and Zika viruses; correspondingly there is a substantial body of work attempting to establish sHEG drives in *Ae. aegypti*. This discussion is especially timely, as 2024 has recorded the highest global dengue case count to date^30^, with climate change projected to exacerbate the situation^31^. While sHEG drives offer a promising strategy to curb the escalating public health burden, their success hinges on achieving consistently high homing efficiency. Without this, their potential will remain an unrealised ambition. Notably, even the best-performing sHEG strains in *Ae. aegypti*^19^ have yet to achieve homing efficiency comparable to *Anopheles*, with additional challenges related to fitness costs.

### Deciphering Homing Efficiency: Biological Constraints or Technological Shortcomings?

The rapid success of sHEG systems in *Anopheles* may have inadvertently set unrealistic expectations for other species. Researchers were motivated to design sHEG systems in other species without apparent need for a comprehensive understanding of the fundamental biology underlying efficient sHEGs. As a result, many sHEG constructs in non-*Anopheles* species replicate the early *Anopheles* core designs (i.e. a germline-active Pol II promoter driving Cas9, sgRNAs targeting a genomic locus, and Pol III promoters for sgRNA expression) under the assumption that similar results could be achieved.

Despite the construction of sHEGs targeting loci such as *kmo, white, Carb109*, and *TIMP-P4*, using multiple sgRNA and Cas9 regulatory elements, inheritance bias in *Ae. aegypti* remains moderate at best. For instance, *sds3*G1-Cas9 combined with *kmo*^sgRNAs^ achieved an average inheritance of 86% in males and 94% in females^19^. By contrast, the first published Cas9-based sHEG systems in *An. gambiae* and *An. stephensi* achieved inheritance rates of >99%^32,33^. This stark difference raises a critical question: is *Anopheles* biologically distinct from *Ae. aegypti* (and other insect species) in its DNA cleavage and repair mechanisms, or are researchers struggling to replicate specific - though unknown - technical features achieved early, and perhaps fortuitously, in *Anopheles* systems? Answering this question is crucial because the solution will depend on where the bottleneck lies—biology, technology, or both.

**Figure 1:**
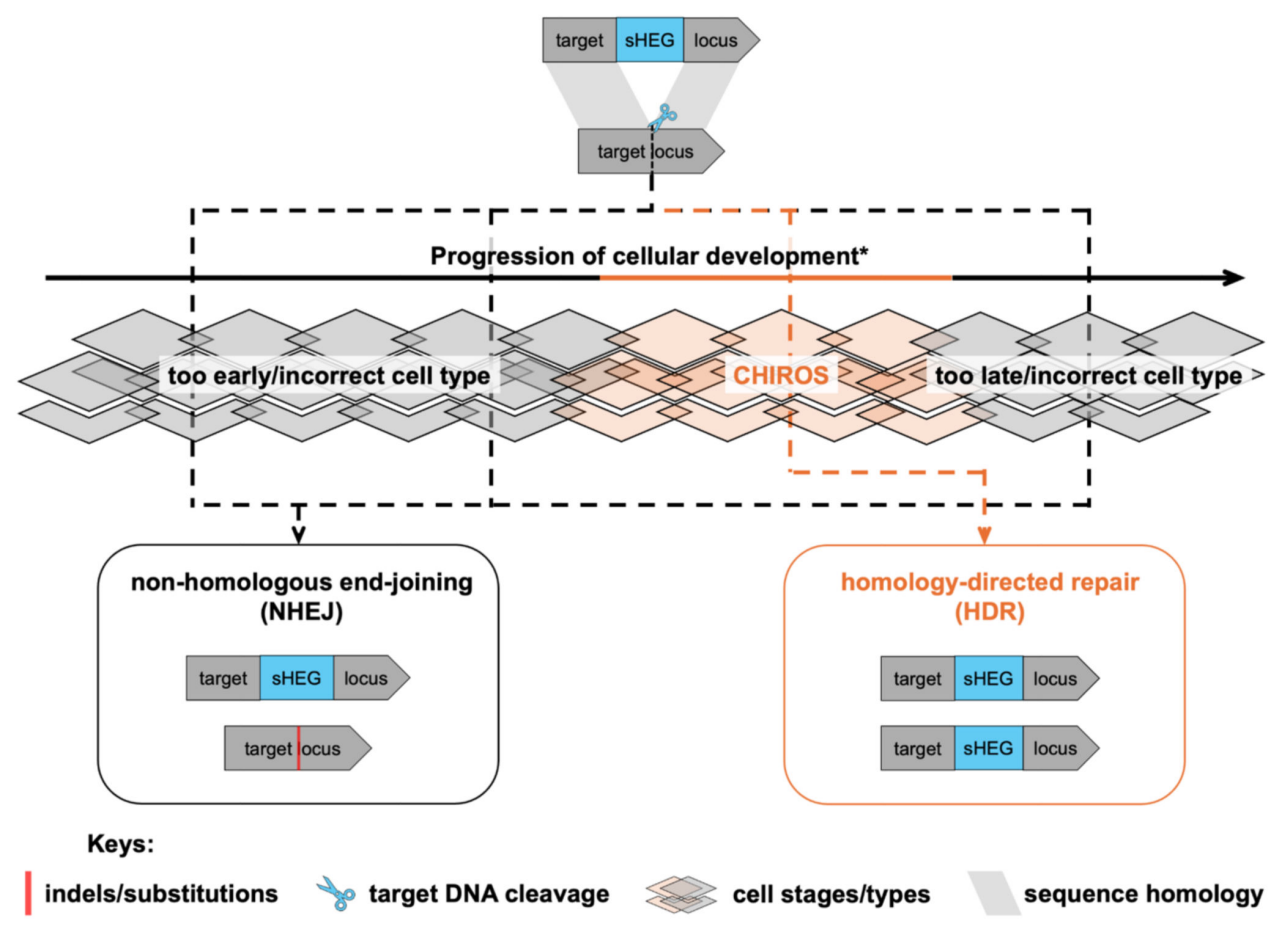
Illustration of the elusive CHIROS (Cell stages where Homing Is the preferred Repair Outcome of Site cleavage). It is hypothesised that homing/HDR is the preferred repair outcome when DNA cleavage occurs during CHIROS, whereas NHEJ is favoured when cleavage takes place in other cells or developmental stages. *Despite the hypothesis that there is a gametogenic window optimal for homing, CHIROS does not necessarily have to be confined to gametogenesis. A number of studies have suggested homing can occur in the embryo, a process termed ‘shadow drive’^41–43^.

**Figure 2:**
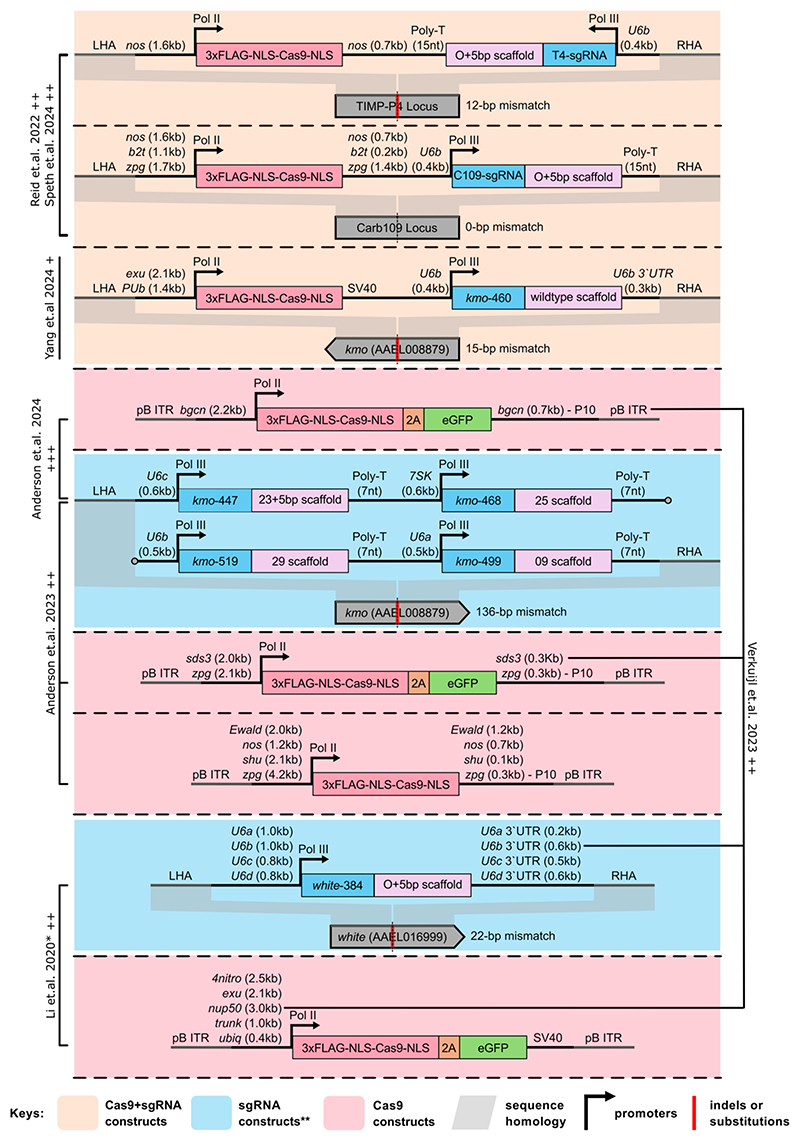
Overview of published *Ae. aegypti* sHEG constructs. Autonomous (both Cas9 and sgRNA within a single construct) and split (Cas9 and sgRNA expressed in separate constructs) designs used to generate *Ae. aegypti* sHEG strains are illustrated. Key abbreviations: pB ITR = piggyBac inverted terminal repeat; LHA = left homology arm; RHA = right homology arm; UTR = untranslated region. Accession IDs for U6 and 7SK genes are as follows: *U6a* (AAEL017702), *U6b* (AAEL017774), *U6c* (AAEL017763), *U6d* (AAEL017905), and *7SK* (AAEL018514). Highest achieved average inheritance rates from each study are indicated by ‘+’ = 50-69%, ‘++’ = 70-89%, ‘+++’ = 90-100%. The O+5bp scaffold was a slightly modified version initially used by Li et al.^22^ to increase sgRNA expression by removing cryptic termination sequences. *Note: Only the *exu*-Cas9 strain was tested in combination with all four *w*^*U6a-d-GDe*^ sgRNA-expressing strains. All other Cas9 strains were assessed only with *w*^*U6b-*GDe^. Additionally, a 276-bp unintended insertion was reported between the 3′ end of the 3′ UTR and RHA in the *w*^*U6d-*GDe^ strain^22^. **Target site naming convention: When a sgRNA target site resides within an exon, it is named using the gene name followed by the nucleotide position of the expected cut relative to the start of the exon (e.g., *kmo*-447).

The prevailing approach to optimising homing efficiency involves inducing DNA cleavage during a hypothetical gametogenic “window” where homing is more likely to occur than end-joining. We refer to this window as CHIROS (Cell stages where Homing Is the preferred Repair Outcome of Site cleavage), drawing inspiration from Kairos, the Greek god of critical moments (Figure 1). Timing of DNA cleavage is thought to be the primary bottleneck in most sHEG systems^34–38^. Cleavage that occurs too early—prior to meiosis I, when homologous chromosomes are physically too far apart—may prevent homing. Conversely, cleavage that occurs post-meiosis I, when homologous chromosomes are no longer present in the same haploid cell, presumably renders homing impossible. Current strategies to exploit this window include using Pol II regulatory elements to restrict Cas9 expression to early gametogenesis and employing Pol III regulatory elements to express sgRNAs. Pol III regulatory elements were primarily derived from U6 and 7SK small nuclear RNA, which are assumed to be constitutively expressed due to their central roles in mRNA splicing and elongation^39,40^. The following is a summary of insights gained from *Ae. aegypti* sHEG construct designs regarding the factors that influence homing efficiency (Figure 2).

#### Cas9 regulatory elements

Of the 13 regulatory elements tested, six (*bgcn, Ewald, nos, sds3, shu*, and *zpg*) were selected because their homologues in other insect species were previously shown to be specifically expressed during early gametogenesis^19,21,23^. The remaining seven elements (*4nitro, beta-tub85D, exu, nup50, PUb, trunk*, and *ubiq*) were chosen for their high expression levels, either constitutively or during later stages of gametogenesis^22–24^. Among these, only *bgcn, nos, sds3, shu, zpg, exu*, and *nup50* produced statistically significant inheritance bias. Interestingly, while *nos* and *zpg* have achieved inheritance bias rates exceeding 95% in *An. gambiae*^4,5^, the achieved rates at the Carb109 locus in *Ae. aegypti* were below 75%^23^. It is worth noting that many of these promoters were originally characterised in *Drosophila melanogaster*, whose gametogenic timeline may differ significantly from that of *Ae. aegypti*^44^. Moreover, very limited information is available regarding germline expression patterns of sHEG transgenes, and none on the precise timing of DNA cleavage events. Another noteworthy observation was the high variability in inheritance bias induced by *sds3*-Cas9 and *shu*-Cas9 inserted at different genomic locations, ranging from levels not significantly different from Mendelian inheritance (50%) to as high as 94%^19^. Taken together, these findings suggest two key points: (a) positional effects likely play a significant role in influencing the precision of expression timing and/or levels and (b) the necessary Cas9 expression patterns/levels for optimal homing may not yet have been successfully recapitulated in *Ae. aegypti* - clearly not for most lines, but, given that relatively few insertions have been assessed, it may be that even the ‘best’ observed are still some way from the best achievable simply from inserting in an optimal genomic location.

#### sgRNA regulatory elements

A total of five Pol III regulatory elements (*U6a-d* and *7SK*) have been tested, either as singleplex or multiplex constructs. Li et al.^22^ conducted a particularly thorough study systematically comparing different U6 regulatory elements integrated into the same locus. They found that the *w*^*U6b-GDe*^ sgRNA strain induced the highest inheritance bias (71%) and exhibited high somatic cutting efficiency (>90%) when combined with *exu*-Cas9. Interestingly, while *U6d* was shown to induce sgRNA expression from an injected plasmid and facilitated HDR-based transgenesis, the *w*^*U6d-GDe*^ sgRNA strain achieved only 53% inheritance bias and 0% somatic cutting in combination with *exu*-Cas9. Another intriguing observation arose when *sds3*G1-Cas9, which achieved up to 94% inheritance bias with *kmo*^sgRNAs^, was combined with *w*^*U6b-GDe*^ sgRNA strain. The latter, which achieved up to 81% inheritance with *nup50*-Cas9, resulted in only a maximum of 67% average inheritance when paired with *sds3*G1-Cas9^20^. These findings suggest that different Pol III promoters likely drive distinct expression patterns and/or levels – there is not necessarily a match between timing of sgRNA expression and that of Cas9 – challenging the conventional assumption that sgRNAs expressed under Pol III promoters are constitutive and highly expressed.

#### Germline DNA cleavage rates

Another potentialexplanation for the low homing efficiency is that *Ae. aegypti* might have intrinsically low germline cutting rates. However, this has been ruled out in studies using *bgcn*-Cas9 and *sds3*G1-Cas9 in combination with *kmo*^sgRNAs^, where germline cut rates were determined to be between 90–100%^19,21^. The observation that not all cleavage events resulted in homing strongly suggests that the timing of DNA cutting, or choice of repair pathway, is the limiting factor, rather than the overall abundance or cutting efficiency of Cas9 and/or sgRNAs.

#### Homology arm sequence heterology

Homing depends on the ability of the cut chromosome to recognise the sHEG-carrying donor chromosome as the HDR template, meaning sequence similarity between the homology arms of the cut and donor chromosomes may influence homing efficiency. Interestingly, all but one construct targeting the C109 locus^23^ exhibited varying degrees of sequence heterology between the cut and donor chromosomes (Figure 2). Constructs with perfect homology arms for these loci were not tested, it remains unclear whether such designs would improve inheritance bias. In a non-homing context, sequence heterology has been shown to negatively affect HDR in *Ae. aegypti*^45^, but results in other insect species in a homing context are contradictory^46–48^.

At this stage, it seems reasonable to conclude that researchers have approached the optimisation of sHEG constructs in *Aedes aegypti* with well-reasoned strategies without any evident flaws in the core designs. While the gradual, albeit enigmatic, improvements in homing efficiency over time suggest there is room for further optimisation, likely through refining the technology, it remains possible that *Ae. aegypti* possesses biological constraints that limit homing efficiency. However, the precise steps required to fully optimise the technology remain uncertain. Notably, recent unexpectedly low drive inheritance in some instances in *Anopheles*, despite employing similar designs, hint that early successes in *Anopheles* may have been partially fortuitous^49–51^. In contrast, efforts in other species appear to have started from a less advantageous position and continue to grapple with significant technological obstacles.

### sHEG Drives in *Ae*. *aegypti*: A Persisting Pursuit Amid Alternatives

Given the current uncertainty regarding the next steps for improvement, one might question whether pursuing sHEGs in *Ae. aegypti* remains worthwhile, especially in light of alternative genetic biocontrol technologies. Established approaches, such as SIT^52^, IIT^53,54^, fs-RIDL^55^, and the *Wolbachia* replacement strategy^56^ have shown promising suppressive effects and/or efficacy in reducing dengue incidence. However, these methods come with their own limitations. Many require repeated mosquito releases, resulting in high deployment costs, or are sensitive to high temperatures^57,58^—both of which pose significant challenges for low- and middle-income countries that are disproportionately affected by *Ae. aegypti*-borne diseases and are located in some of the hottest regions in the world^31^. To address these challenges, gene drive systems must be developed alongside existing strategies to ensure the availability of affordable and equitable solutions when they are needed. However, it is important to note that sHEG drives are not the only gene drive systems under consideration.

For population suppression, the Y-linked X-shredder strategy represents a compelling alternative to synthetic sHEGs. This approach biases the inheritance of the Y chromosome by shredding the X chromosome in the male germline prior to sperm maturation, ultimately leading to male-biased populations and population collapse^8,59,60^. While promising, this strategy presents technical challenges, as it requires the expression of Cas9 and sgRNAs from the Y chromosome during spermatogenesis—a stage where the Y chromosome is often silenced^61^. In culicine mosquitoes, where sex determination is controlled by a heterologous male-determining locus on an autosome rather than heteromorphic sex chromosomes, this obstacle may be less pronounced. However, unlike in XY systems, identifying female-specific locus sequences for targeted shredding poses an additional challenge. At this stage, the feasibility of a Y-linked X-shredder in *Ae. aegypti* remains speculative.

For population modification, toxin-antidote systems such as Cleave and Rescue (ClvR)^62,63^ and Toxin Antidote Recessive Embryos (TARE)^64^ offer alternative gene drive approaches. In these systems, Cas9+sgRNA targeting an essential gene acts as a toxin, while a cleavage-resistant rescue construct serves as the antidote. Unlike sHEGs, these systems do not increase the inheritance of a favourable genetic trait by replicating it. Instead, they rely on inducing a significant fitness cost in individuals that do not carry the antidote. ClvR and TARE have been successfully implemented in *D. melanogaster* and *Arabidopsis thaliana*^65^, but the design of these systems makes them difficult to adapt for population suppression, in addition to spreading more slowly than homing-based systems. While not impossible, as demonstrated by Champer et al.^66^, such systems tend to be either less robust or technically challenging to construct.

One notable advantage of both the Y-linked X-shredder and ClvR/TARE systems over sHEGs is that they depend only on cutting, rather than cutting and homing, during germline development. Given that very high cleavage rates have already been demonstrated in *Ae. aegypti*, this aspect should not pose a significant obstacle. Nonetheless, sHEGs continue to hold significant promise as a genetic biocontrol tool due to their inherent flexibility - once optimised, they could be readily adapted for both population suppression and modification - and more rapid spread. This adaptability offers stakeholders, particularly communities where the technology is deployed, the ability to choose either strategy based on their needs. Furthermore, efforts to refine sHEG technology have the potential to provide valuable insights into germline HDR mechanisms, thereby deepening our understanding of fundamental biological processes.

### CHIROS: The Key to Systematic Advancements in sHEG Development

The likelihood of sHEGs succeeding in non-*Anopheles* species such as *Ae. aegypti* depends on whether rational design can be systematically applied to optimise sHEGs for this species. The persistent inability to identify the factors that drive successful or poor homing efficiency has significantly impeded progress, making advancements slow and frustrating. This leaves researchers navigating a trial-and-error process without a clear understanding of what changes might lead to meaningful improvements. Without addressing these uncertainties, further efforts to optimise homing efficiency will likely remain unfocused and ineffective.

To move forward, the field may need to break free from the cycle of iteratively “fixing” constructs, focusing more on first understanding the underlying problem(s). A first priority should be to determine whether CHIROS exists. Fortunately, a sufficient number of drive strains have already been developed, enabling researchers to revisit these established strains and conduct targeted experiments to address this question. Spatial detection of Cas9 mRNA, Cas9 protein, and sgRNA in gonadal tissue could help infer the presence of CHIROS, particularly if co-localisation of Cas9 and sgRNA correlates with specific germline stages and higher or lower homing efficiency. A meta-analysis of all published sHEG systems could further identify factors most strongly associated with homing success. In parallel, the development of reporter assays^67^ capable of detecting homing events in the gonads would be valuable, as these tools could offer direct insights into the timing and cellular context of homing.

If CHIROS can be identified and characterised, the path to rational improvement becomes clearer. Synthetic biology tools that enable precise temporal and spatial expression of Cas proteins and sgRNAs would help overcome current technological limitations in non-*Anopheles* species^68^. Notably, the recent publication of a high-resolution single-nucleus transcriptomic atlas^69^ for *Ae. aegypti* provides an unprecedented opportunity to mine for new germline regulatory elements that could facilitate expression within CHIROS. Conversely, if CHIROS does not exist, alternative strategies should be considered. One possible direction is the use of Cas9 fusion proteins engineered to enhance homology-directed repair—an approach that may improve homing efficiency regardless of CHIROS’s existence^70^. Through these fundamental and technological advancements, the field may finally unlock the full potential of sHEG drives in *Ae. aegypti*, realising their promise as a powerful and equitable tool for vector control and public health.

## Data Availability

No data were generated for the research described in the article.
